# Errata

**DOI:** 10.4269/ajtmh.2011.851err

**Published:** 2011-07-01

In *Am J Trop Med Hyg 84:* 653–661 by Marzouki et al, there was an error in the published version of Figure 2D. The correct version appears here.

**Figure 2:**
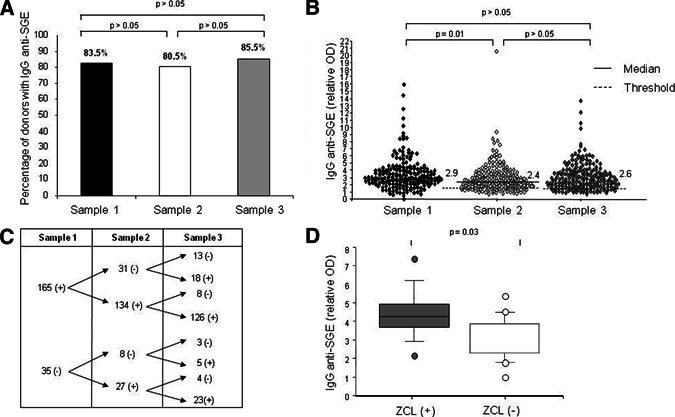


In *Am J Trop Med Hyg 84:* 753–756 by Higazi et al, the fourth author's name is listed incorrectly. The correct name should be Wigdan A. Elmubark, not Wigdan A. Mohamed.

In *Am J Trop Med Hyg 84:* 851–857 by Inglis et al, the last author's middle initial was omitted. The author's name should be Christopher H. Heath.

In *Am J Trop Med Hyg 84:* 862–869 by Reyburn et al, an incorrect unit of measurement appears in the print version of the article. It should say “… an increase of 200 mm” in the sixth line of the abstract and on page 865 in the second column, line six.

